# College of Physicians and Surgeons

**Published:** 1886-04

**Authors:** 


THE COLLEGE OF PHYSICIANS AND SURGEONS OF CHICAGO.—The Annual Commencement of the College of Physicians and Surgeons of Chicago was held on Tuesday, February 23. Degrees were awarded to 71 members of the same class, which numbered 85. During the winter session there were 165 matriculates. Dr. E. F. Wells, of Minster, Ohio, has been elected Professor of Materia Medica.

PROF. E. QUINE has resigned the chair of Practice of Medicine.

